# Cap0037, a Novel Global Regulator of *Clostridium acetobutylicum* Metabolism

**DOI:** 10.1128/mBio.01218-16

**Published:** 2016-10-04

**Authors:** Ngoc-Phuong-Thao Nguyen, Sonja Linder, Stefanie K. Flitsch, Bettina Schiel-Bengelsdorf, Peter Dürre, Philippe Soucaille

**Affiliations:** a LISBP, Université de Toulouse, CNRS, INRA, INSA, Toulouse, France; b Metabolic Explorer, Biopôle Clermont-Limagne, Saint-Beauzire, France; c Institute of Microbiology and Biotechnology, University of Ulm, Ulm, Germany

## Abstract

An operon comprising two genes, *CA_P0037* and *CA_P0036*, that encode proteins of unknown function that were previously shown to be highly expressed in acidogenic cells and repressed in solventogenic and alcohologenic cells is located on the pSOL1 megaplasmid of *Clostridium acetobutylicum* upstream of *adhE2*. A *CA_P0037*::*int* (*189/190s*) mutant in which an intron was inserted at position 189/190 in the sense strand of *CA_P0037* was successfully generated by the Targetron technique. The resultant mutant showed significantly different metabolic flux patterns in acidogenic (producing mainly lactate, butyrate, and butanol) and alcohologenic (producing mainly butyrate, acetate, and lactate) chemostat cultures but not in solventogenic or batch cultures. Transcriptomic investigation of the *CA_P0037*::*int* (*189/190s*) mutant showed that inactivation of *CA_P0037* significantly affected the expression of more than 258 genes under acidogenic conditions. Surprisingly, genes belonging to the Fur regulon, involved in iron transport (*CA_C1029-CA_C1032*), or coding for the main flavodoxin (*CA_C0587*) were the most significantly expressed genes under all conditions, whereas *fur* (coding for the ferric uptake regulator) gene expression remained unchanged. Furthermore, most of the genes of the Rex regulon, such as the *adhE2* and *ldhA* genes, and of the PerR regulon, such as *rbr3A-rbr3B* and *dfx*, were overexpressed in the mutant. In addition, the whole *CA_P0037-CA_P0036* operon was highly expressed under all conditions in the *CA_P0037*::*int* (*189/190s*) mutant, suggesting a self-regulated expression mechanism. Cap0037 was shown to bind to the *CA_P0037-CA_P0036* operon, *sol* operon, and *adc* promoters, and the binding sites were determined by DNA footprinting. Finally, a putative Cap0037 regulon was generated using a bioinformatic approach*.*

## IMPORTANCE

*Clostridium acetobutylicum* is well-known for its ability to produce solvents, especially *n*-butanol. Understanding the regulatory network of *C. acetobutylicum* will be crucial for further engineering to obtain a strain capable of producing *n*-butanol at high yield and selectivity. This study has discovered that the Cap0037 protein is a novel regulator of *C. acetobutylicum* that drastically affects metabolism under both acidogenic and alcohologenic fermentation conditions. This is pioneering work for further determining the regulatory mechanism of Cap0037 in *C. acetobutylicum* and studying the role of proteins homologous to Cap0037 in other members of the phylum *Firmicutes*.

## INTRODUCTION

*Clostridium acetobutylicum* is a Gram-positive, strictly anaerobic, spore-forming bacterium now considered the model organism for the study of solventogenic clostridia (1, 2). The superiority of butanol over ethanol as an alternative biofuel has attracted research interest in *C. acetobutylicum* and other recombinant bacteria producing butanol as a major product (3). Understanding the complex regulatory network of *C. acetobutylicum* metabolism is crucial for further manipulating the genotype to obtain industrial strains, but our understanding of cellular functioning remains very limited. Some global regulators have already been studied. A peroxide repressor (PerR)-homologous protein was identified to be a key repressor that plays an important role in defense against oxidative stress (4, 5). In the same family as PerR, a ferric uptake regulator (Fur), which helps *C. acetobutylicum* sense and respond to iron availability at multiple levels using a sophisticated system, was identified and well characterized (6). Spo0A was found to directly control the solventogenic switch, expression of the *adc* and *ptb* genes (7), and activation of the sporulation program in *C. acetobutylicum* (8). The carbon storage regulator (CsrA) was also shown to directly or indirectly regulate multiple pathways, including flagellum assembly, oligopeptide transport, iron uptake, phosphotransferase systems (PTS), synthesis of riboflavin, stage III sporulation, and central carbon metabolism (9). Recently, a redox-sensing transcriptional repressor (Rex) has been found to modulate its DNA binding activity in response to the NADH/NAD^+^ ratio and plays a role in the alcohologenic shift of *C. acetobutylicum* (10, 11).

**FIG 1:**
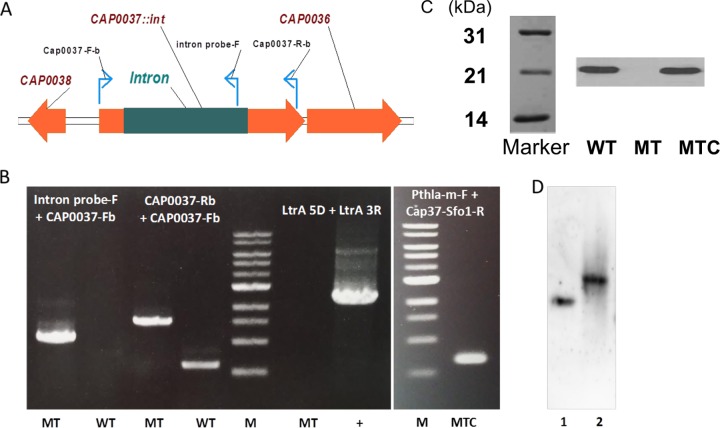
Construction of *CA_P0037*::*int* mutants. (A) DNA configuration of mutant with a group II intron inserted at position 189/190 on the sense strand of *CA_P0037*. (B) PCR on genomic DNA of *CA_P0037*::*int* mutant (MT) and wild-type (WT) strains with different pairs of primers (intron probe-F and CAP0037-Fb primers and CAP0037-Rb and CAP0037-Fb primers) to confirm intron insertion and primers LtrA 5D and LtrA 3R to demonstrate the loss of pCUI1-cap37(189s). PCR was performed on genomic DNA of complemented mutant (MTC) strain with primers pthla-MF and cap37-sfo1-R to demonstrate the presence of the pSOS95-Cap0037 plasmid. (C) Detection of the Cap0037 protein in the wild type (WT), *CA_P0037*::*int* mutant (MT), and *CA_P0037*::*int* complemented mutant (MTC). The Western blot was generated using polyclonal antibodies raised against purified His-tagged Cap0037. The positions of molecular mass markers (in kilodaltons) are shown to the left of the Marker blot. (D) Southern blot to demonstrate single intron insertion in the *CA_P0037*::*int* mutant. The intron probe was DIG labeled and hybridized to EcoRI-digested genomic DNA of the *CA_P0037*::*int* mutant (lane 1) and to the AgeI-SbfI-digested pCUI1-cap37(189s) plasmid as a positive control (lane 2) with expected sizes of 4,388 bp and 1,718 bp, respectively.

The regulatory network of *C. acetobutylicum* remains largely unexplored. In this study, we found Cap0037 to be a novel self-regulated protein that globally affects *C. acetobutylicum* metabolism. *CA_P0037* is the first gene of a polycistronic operon with *CA_P0036* (Fig. 1). This operon is located upstream of *adhE2* on the pSOL1 megaplasmid, and it was significantly expressed in acidogenic cells and repressed in solventogenic and alcohologenic cells (12, 13). Furthermore, the two proteins were present at a 1:1 molar ratio and were among the 30 most abundant proteins in the cell (around 50,000 molecules per cell) (12). Another study found that *CA*_*P0037*-*CA*_*P0036* expression was slightly downregulated in an *adc* mutant, clearly upregulated during solventogenesis in a *pta* mutant and an *adc pta* double mutant, and strongly repressed in a *ptb* mutant independent of the cultivation conditions (14). Interestingly, Cap0037 and Cap0036 were found to appear in multiple spots (up to five in the case of Cap0037 and up to at least four in the case of Cap0036) in a two-dimensional (2-D) gel analysis of acidogenic cells, suggesting posttranslational modifications, such as phosphorylation, acetylation, glycosylation, or methylation (13).

In order to investigate the roles of these proteins in the acidogenic state of *C. acetobutylicum*, we inactivated *CA_P0037* via the mobile group II intron. The regulatory role of Cap0037 and the observed phenotypes will be discussed in light of the different -omic data sets. DNA footprinting was used to determine DNA binding motifs of Cap0037 on its own promoter and in the upstream region of selected genes shown to be up- or downregulated. Putative Cap0037 binding sites across *C. acetobutylicum* genomes were analyzed via a bioinformatic approach. This study builds a foundation to further investigate the regulatory mechanism of this novel protein in *C. acetobutylicum*, which should be beneficial for understanding the role of homologous proteins in other members of the phylum *Firmicutes*.

## RESULTS AND DISCUSSION

### Phylogenetic tree and bioinformatic analysis of Cap0037/Cap0036.

Cap0037 and Cap0036 are proteins of unknown function. They are well conserved in other *Firmicutes* such as *Clostridium*, *Bacillus*, and *Geobacillus* (see Fig. S1 in the supplemental material). The functions of these two conserved proteins have not been characterized.

Cap0036 was found to be homologous to some cytoplasmic proteins of the *Bacillus* species and also to some bactofilin family proteins from the *Geobacillus* species (see Fig. S1A in the supplemental material). However, no transmembrane domains could be identified in Cap0036 using TMpred (15) and OCTOPUS (16), indicating that Cap0036 is most probably a cytoplasmic protein, rather than a membrane protein of the bactofilin family.

Noticeably, Cap0037 is homologous to the YgaS-like protein in *Bacillus cereus* G9241 (a putative DNA-binding protein), although the relationship is distant (see Fig. S1B in the supplemental material). A helix-turn-helix DNA binding motif (ISKKELLEITHISYGQLYRWKR) was found in the N terminus of the Cap0037 protein sequence (by the NPS@ web-based biotool [17]). As a result, Cap0037 is likely a novel transcriptional regulator. The 1:1 expression ratio of Cap0037 and Cap0036 (12) suggested an interaction of these two proteins that forms a system acting as an unknown regulatory mechanism in *C. acetobutylicum*. Interestingly, a transmembrane domain (LIRKLGIAICFLMLIPNEIYI) and an outer membrane region (ENTASLVLKVNARECLEELKSKLSL) were found at the C terminus of Cap0037 (OCTOPUS program [16]).

In this study, *CA_P0037* was inactivated, and the effect of *CA_P0037* disruption was elucidated at the system level.

### Disruption of *CA_P0037* by pCUI-Cap0037 (189s).

The ClosTron technique was first applied (18, 19) to inactivate *CA_P0037* targeting an insertion into the 277/278 antisense of the *CA_P0037* coding sequence (CDS), but targeted intron insertion was not detected in all of the erythromycin-resistant clones obtained. Therefore, we used another intron-based method developed in our laboratory (20) for knocking out *CA_P0037* based on the pCUI-cap0037 vector as mentioned in Materials and Methods. The mutant was successfully obtained with the use of plasmid pCUI-Cap0037: a 1-kbp-long intron was inserted at position 189/190 in the sense strand of *CA_P0037*. PCR using two external primers flanking the targeted position confirmed the insertion of the intron into the gene (Fig. 1B). After selecting for the *CA_P0037*::*int* mutant, the strain was subjected to 5′-fluorouracil (5′-FU) selection to cure the pCUI-Cap0037 plasmid to avoid intron splicing by LtrA (the LtrA-encoding gene is present on the plasmid backbone). Loss of the plasmid was validated by sensitivity to erythromycin and by PCR (Fig. 1B). Southern blotting was conducted to confirm a single intron insertion into the genome (Fig. 1D).

Quantitative transcriptional analysis of the *CA_P0037*::*int* mutant under acidogenic chemostat culture conditions showed that the numbers of *CA_P0037* and *CA_P0036* mRNA molecules per cell (88 and 76, respectively) were similar to those in the wild-type (WT) strain (78 for both), indicating that the intron insertion has no polar effect and does not affect the stability of the transcript. Such results were expected as, in contrast to the ClosTron, the short intron that was inserted does not contain any terminator of transcription. On the other hand, when the level of Cap0037 protein was measured by Western blotting under acidogenesis conditions, no protein was detected for the *CA_P0037*::*int* mutant (Fig. 1C). However, when the *CA_P0037*::*int* mutant was transformed with the pSOS95-Cap0037 plasmid to express the *CA_P0037* gene under control of the *thlA* promoter under acidogenesis conditions, both the production of Cap0037 and the phenotype (as will be seen below) were restored.

### Carbon and electron fluxes of the *CA_P0037*::*int* mutant under different physiological conditions.

Acidogenesis and alcohologenesis were the two states that were significantly changed in the *CA_P0037*::*int* mutant. The normalized metabolic fluxes were compared to previously published data for the control strain (12).

**FIG 2:**
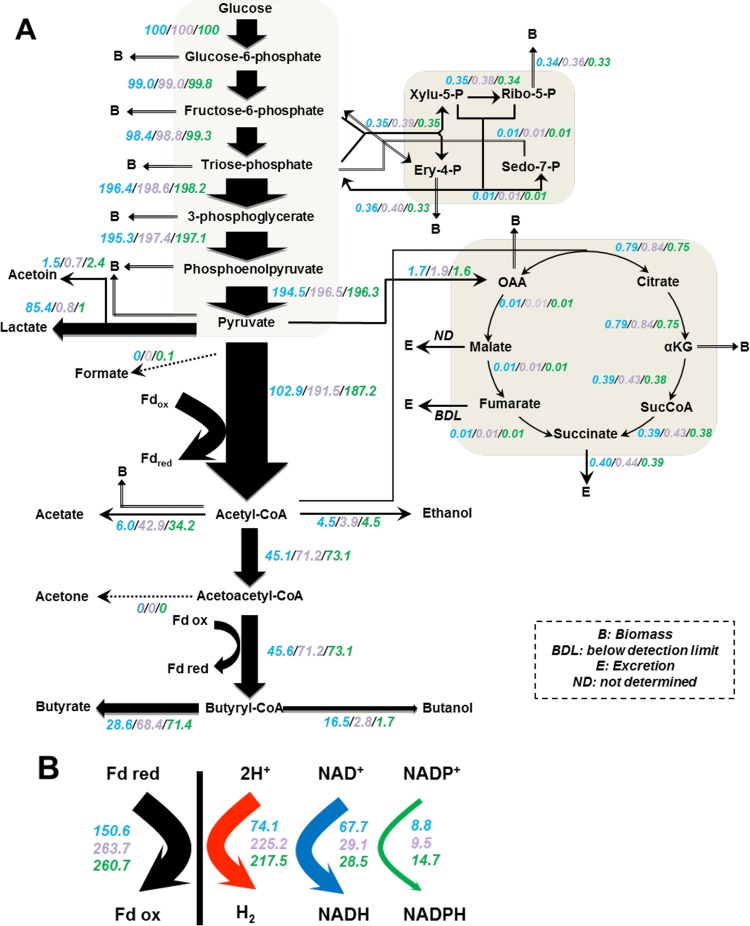
Metabolic fluxes of the *CA_P0037*::*int* mutant and complementary strain versus control strain under acidogenesis conditions. (A) Carbon fluxes; (B) electron flux. All values are normalized to the flux of the initial carbon source (millimoles per gram [dry cell weight] per hour). Glucose flux is normalized and set at 100. The values of the different strains are shown in color as follows: the corresponding mutant in blue, the complementary strain in purple, and the control strain in green. The control data were from reference 12. Metabolic fluxes of the *CA_P0037*::*int* mutant versus control strain under alcohologenesis and solventogenesis conditions can be found in the supplemental material. Abbreviations: Fd_ox_ and Fd_red_, oxidized and reduced ferredoxin, respectively; OAA, oxaloacetate; αKG, α-ketoglutarate.

In the acidogenic condition, the mutant strain underwent a profound change in its metabolism: the normalized fluxes of butyrate and acetate decreased 2.5- and 5.7-fold, respectively, while the normalized fluxes of lactate and butanol increased 85- and 9.7-fold, respectively (Fig. 2A). The normalized fluxes of ethanol, on the other hand, remained unchanged. In a complementation experiment, the *CA_P0037*::*int* mutant was transformed with the pSOS95-Cap0037 plasmid to express the *CA_P0037* gene under control of the *thlA* promoter. When this strain was evaluated in acidogenic chemostat culture in the presence of clarithromycin (40 µg/ml) for the maintenance of the plasmid, the normalized metabolic fluxes (Fig. 2A) were not significantly different from those of the wild-type strain (12), demonstrating that the *CA_P0037*::*int* mutant phenotype was attributable only to *CA_P0037* inactivation. The production of butanol by the *CA_P0037*::*int* strain under acidogenesis conditions is explained by the higher *adhE2* expression (~50-fold higher than the control strain, with 21 mRNA molecules/cell) (12) (see Data Set S1 in the supplemental material), and although the expression of the *sol* operon (*ctfA*, *ctfB*, and *adhE1*) was increased ~6-fold, the number of mRNA molecules remained low (0.5 mRNA molecule/cell) compared to *adhE2* (21). The increase in lactate formation was associated with an 83-fold increase in *ldhA* expression (12) (Data Set S1). For the *CA_P0037*::*int* mutant, the acetate flux decreased 5.7-fold compared to the control strain, although *pta-ack* (*CA_C1742–CA_C1743*) expression was unchanged (Data Set  S1). Thus, flux is controlled at the enzyme level via a decrease in the acetyl coenzyme A (acetyl-CoA) pool, probably due to the twofold-higher expression of most of the genes (*CA_C2708–CA_C2712*) coding for the enzymes converting acetoacetyl-CoA to butyryl-CoA.

The alcohologenesis state (pH 6.3, in a mixture of glucose and glycerol) was the second most affected state following the disruption of *CA_P0037* (see Fig. S2 in the supplemental material). Although all of the glucose provided in the medium was consumed, the normalized flux of glycerol consumption was reduced more than 15-fold compared to the control strain. While the control strain produced mainly ethanol (normalized flux of 8.6%), butyrate (normalized flux of 25.1%), and butanol (normalized flux of 61.1%), the mutant produced mainly acids: butyrate (normalized flux of 63.6%), acetate (normalized flux of 21.9%), and lactate (normalized flux of 11.8%) (Fig. S2A). The ethanol and butanol fluxes were 1.8-fold and 9.8-fold lower than those in the parental strain, respectively, suggesting that, in the mutant, the alcohologenesis state was barely induced, and it behaved like the control strain in the acidogenesis state. The lower glycerol consumption of the *CA_P0037*::*int* strain is explained by the lower expression (four- to sixfold decrease) of the gene cluster coding for glycerol transport and utilization (*CAC1319–CAC1323*). The increase in lactate formation was associated with a 32-fold increase in *ldhA* expression, and the lower production of butanol was associated with 10-fold-lower *adhE2* expression. On the other hand, the butyrate flux increase was not associated with a change in the expression of *ptb-buk* (*CA_C3074–CA_C3075*), which remained unchanged (12) (Data Set  S1). Thus, flux is controlled at the enzyme level via an increase in the butyryl-CoA pool, probably due to the 10-fold-lower level of expression of *adhE2*.

The carbon flux of the mutant strain under solventogenesis conditions was compared to previously published data for the control strain. The *CA_P0037*::*int* mutant was not able to consume all glucose, and therefore, the titers of products were lower, but the fluxes were not significantly different from those of the wild-type strain (see Fig. S2B in the supplemental material). The level of expression of both the *sol* operon and the *adhE2* gene were higher in the mutant (two- and fourfold, respectively), although the number of mRNA molecules for *adhE1* (12 molecules/cell) was much higher than for *adhE2* (~1 molecule/cell). Surprisingly, the expression of *adc* was downregulated threefold in the mutant, although the number of mRNA molecules per cell (4.3) remained high.

The normalized electron fluxes were analyzed for the *CA_P0037*::*int* mutant under three different conditions. In acidogenesis, the primary use of reduced ferredoxin was switched from hydrogen to NADH production in response to the high expression of *adhE2* and *ldhA* in the mutant (Fig. 2B). In this metabolic state, the normalized hydrogen production fluxes decreased ~3-fold, while the normalized fluxes of NADH production from reduced ferredoxin increased 2.4-fold. The decrease in hydrogen production was not attributable to the expression of *hydA* (*CA_C0028*), which remained unchanged, but to the lower flux of reduced ferredoxin production, as due to high *ldhA* expression, lactate became the major product. The 2-fold increase in the normalized ferredoxin-NAD^+^ reductase fluxes might be explained by the 500-fold-higher expression of *CA_C0587* encoding the major flavodoxin and the 1.5-fold-lower expression of *CA_C0303* encoding the major ferredoxin, as well as the possibility that reduced flavodoxin might be a better substrate than ferredoxin for the ferredoxin-NAD^+^ reductase. In solventogenesis, the normalized electron fluxes were not significantly changed in agreement with the conserved product pattern (see Fig. S2D in the supplemental material). In alcohologenesis, butyrate was the main fermentation product of the *CA_P0037*::*int* mutant, and the normalized hydrogenase flux increased 1.4-fold, while the normalized ferredoxin-NAD^+^ reductase fluxes decreased 2.6-fold (Fig. S2C). The high normalized hydrogen production and low normalized ferredoxin-NAD^+^ reductase fluxes and normalized butanol fluxes can be explained by the fivefold downregulation of *CA_C3486* encoding a potential multimeric flavodoxin and the proposed physiological role (12) in alcohologenic culture of the wild-type strain, providing a better substrate for the ferredoxin NAD^+^ reductase than for hydrogenase.

### Determination of the Cap0037 DNA binding site.

**FIG 3:**
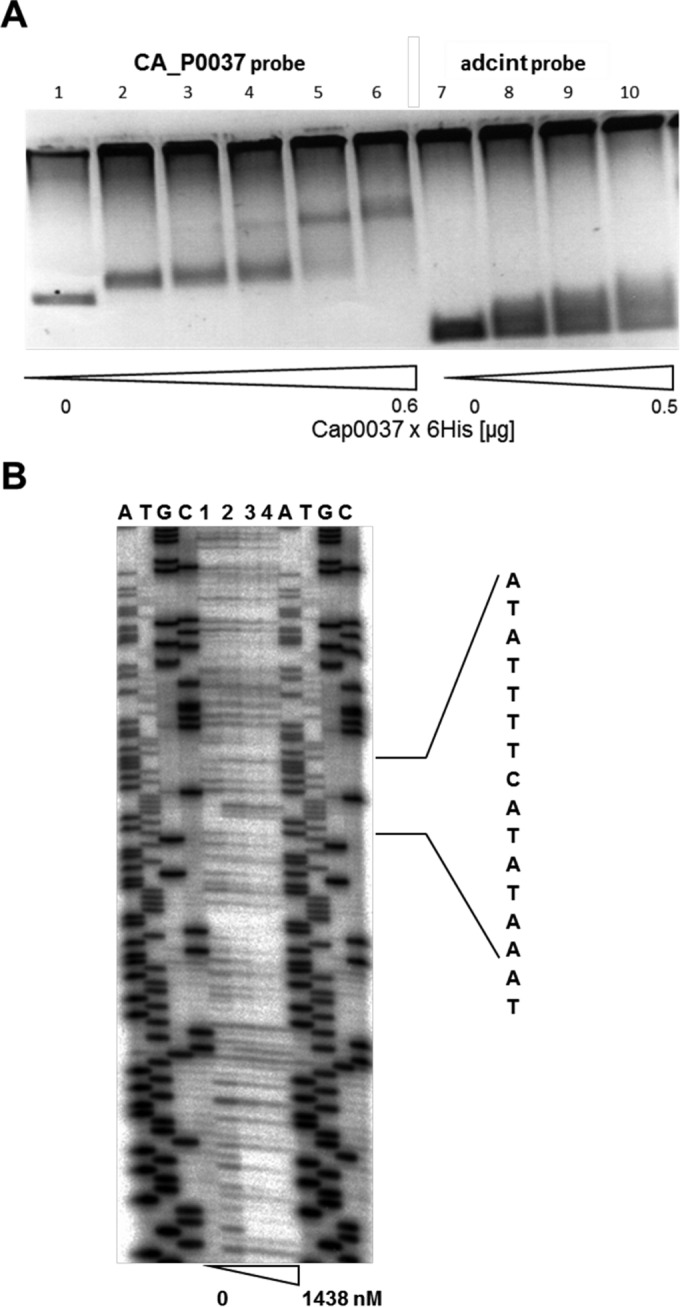
Analysis of Cap0037 binding to the promoter region of the *CA_P0037-CA_P0036* operon. (A) EMSAs using the promoter region of the *CA_P0037-CA_P0036* operon and Cap0037 protein. Lanes 1 to 6, 0, 0.1, 0.2, 0.3, 0.5, and 0.6 µg protein, respectively; lanes 7 to 10, 0, 0.1, 0.2, and 0.5 µg protein, respectively. (B) DNase I protection assay (DNA footprinting) of Cap0037 interacting with the *CA_P0037/CA_P0036* promoter region (probe 85). End-labeled DNA fragment carrying the promoter region of the *CA_P0037-CA_P0036* operon was incubated with different concentrations of Cap0037, subjected to DNase I cleavage, and analyzed on a sequencing gel. Sequencing reaction was performed with plasmid pDrive_86kurz. Lanes 1 to 4 contain 0 to 1,438 nM Cap0037. The assigned region on the right side indicates the region protected by Cap0037 and the respective sequence.

To study the possible regulatory function of Cap0037 in central metabolism, we analyzed the ability of Cap0037 to bind to the promoter regions of several genes coding for key enzymes of the central metabolism of *C. acetobutylicum* that were either upregulated (*CA_P0037/CA_P0036*, *ldhA*, the *sol* operon, and *fld1* [*CA_C0587*]) or downregulated (*adc*) in the *CA_P0037*::*int* mutant under acidogenesis conditions. A shift was observed only for the promoter regions of *CA_P0037/CA_P0036* (Fig. 3A), the *sol* and *adc* operon (see Fig. S3 in the supplemental material), indicating that the upregulation of *ldhA* and *fld1* (*CA_0587*) was an indirect effect of *CA_P0037* inactivation (data for *ldhA* and *fld1* promoters are not shown).

**FIG 4:**
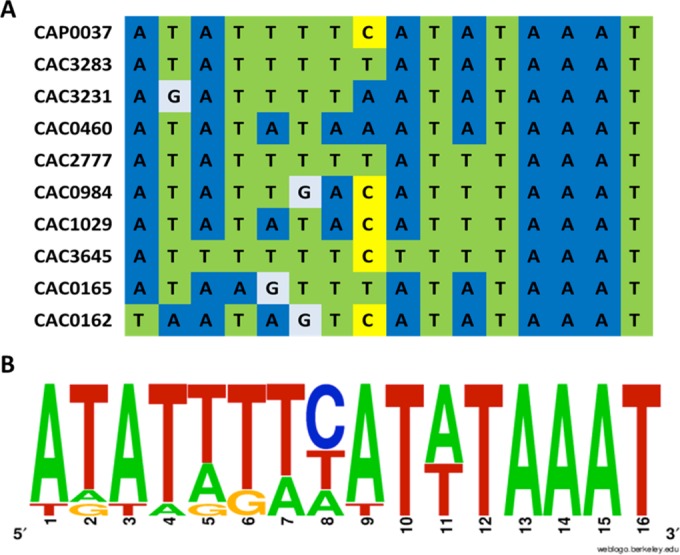
Alignment of putative Cap0037 binding sequence (A) and sequence logo showing the frequencies of residues (B).

**FIG 5:**
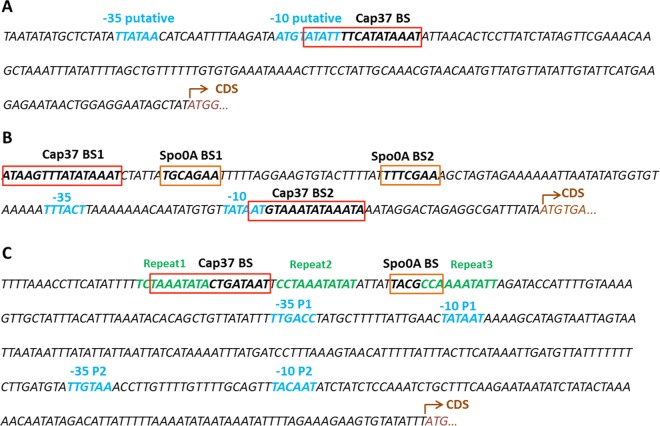
DNA sequence of the 5′ flanking region of *CA_P0037* (A)**,** *adc* (B), and *sol* operon (C). Cap0037 binding sites (Cap37 BS) determined in this study are shown in red boxes. Spo0A binding sites (Spo0A BS) are shown in orange boxes (7, 32). Promoter −35 and −10 elements are shown in blue (7, 33). Putative −35 and −10 sequences of the CA_P0037-CA_P0036 operon were analyzed by the BPROM tool. Three imperfect repeats in the *sol* promoter are shown in green letters (7). Coding sequences (CDS) are shown in brown.

We used DNA footprinting to determine the DNA binding sites (BSs) of Cap0037 in the promoter regions of *CA_P0037* (Fig. 3B) and in the *sol* operon and *adc* gene (see Fig. S4 in the supplemental material). Interestingly, Cap0037 was found to bind to the 5′-ATATTTTCATATAAAT-3′ sequence which overlaps the putative −10 region of the *CAP0037*-*CAP0036* operon promoter (determined by the BPROM tool [21]), suggesting that Cap0037 might repress its own transcription. This hypothesis is in agreement with the derepression (50.7- and 68.6-fold higher than the levels of expression of the control) of the *CA*_*P0037*-*CA*_*P0036* operon observed under alcohologenesis and solventogenesis conditions of the *CA_P0037*::*int* mutant. Two binding sites were found in the *adc* promoter region (Fig. S4A and B). The first was 5′-ATAAGTTTATATAAAT-3′, located upstream of −35, which contains three mismatches compared to the binding site (Fig. 4) found in the *CA_P0037* promoter (Fig. 5). Cap0037 could potentially bind to this sequence to activate the expression of *adc*, which fit with the transcriptomic data for the *CA_P0037*::*int* mutant, in which *adc* was downregulated under all conditions. The second binding site in the *adc* promoter region, 5′-TAATGTAAATATAAAT-3′, is less conserved compared to the *CAP0036*-*CAP0037* operon promoter binding with five mismatches, and it is located between the −10 and +1 promoter region (Fig. 5), suggesting that it might also be involved in the repression of *adc* under conditions that have not been characterized yet. It is worth noting that a Spo0A~P binding box was found upstream of the −35 region of the *adc* promoter (7). This box is only 6 bp downstream of the first Cap0037 binding site (Fig. 5). Hence, there might be some synergy between Spo0A~P and Cap0037 for the activation of *adc*.

In the *sol* promoter region, Cap0037 binds to the 5′-TAAATATACTGATAAT-3′ sequence located upstream of the Spo0A~P binding box and the −35 region of the *sol* operon promoter (Fig. 5). Interestingly, this Cap0037 binding site overlapped the R1 sequence (5′-TCTAAATATA-3′) that has been proposed (22) to be the binding site of an additional activator protein, which acts in concert with Spo0A~P to activate the transcription of the *sol* operon. Binding of Cap0037 would prevent the binding of this activator and would explain the upregulation of the *sol* operon in the *CA_P0037*::*int* mutant in acidogenesis and solventogenesis (see Data Set S1 in the supplemental material).

Using these experimentally validated binding sequences, we can search for putative Cap0037 binding sites across the genome using the RegPredict tool.

### Determination of putative Cap0037 binding sites across the *C. acetobutylicum* genome.

The 5′-ATATTTTCATATAAAT-3′ sequence and the conserved motif were used as references for the construction of a putative Cap0037 regulon by whole *C. acetobutylicum* genome analysis using RegPredict. The sequences with scores higher than 5.0 (which allow three mismatches or less) were selected and further analyzed for their relative position to the CDS of the corresponding gene. In addition to the three Cap0037 binding sites previously identified in the electrophoretic mobility shift assays, eight other putative Cap0037 binding sites were discovered (see Table S2 in the supplemental material). Of these eight sites, the transcription of the genes potentially regulated by Cap0037 binding was analyzed for the *CA_P0037*::*int* mutant and compared to the wild type in the three metabolic states.

The first putative Cap0037 binding site was located between the −35 and −10 promoter regions of an operon (*CA_C1029-CA_C1032*) encoding an iron transport system of the Feo family. This operon was strikingly upregulated under all conditions in the *CA_P0037*::*int* mutant. Previously, this operon was shown to be controlled by Fur (encoded by *CA_C1682*), the ferric uptake regulator, and was found to be highly induced by iron limitation or in the *fur*::*int* mutant (6). There is one Fur box located downstream of the −10 promoter region of *CA_C1029-CA_C1032* (6). However, expression of the Fur-encoding gene was unchanged in the *CA_P0037*::*int* mutant under all continuous culture conditions. Thus, Cap0037 might be the second repressor of this iron transport system, raising the question of how Cap0037 and/or Cap0037 together with Fur carry out a regulatory role.

The second putative Cap0037 binding site was located 7 bp upstream of the −35 promoter region of an operon (*CA_C2777-CA_C2778*) encoding a glutaredoxin and a rubredoxin. This operon was upregulated under all conditions in the *CA_P0037*::*int* mutant. Previously, this operon was shown to be controlled by PerR (encoded by *CA_C1682*), the peroxide repressor, and was found to be induced by oxygen exposure or in a Δ*perR* mutant (5). There is one PerR box located downstream of the −10 promoter region of the *CA_C2277-CA_C2278* operon. Thus, in addition to the iron transport discussed above, which could be regulated by the Cap0037 and Fur proteins, we have potentially identified another operon that can be controlled by two regulators, Cap0037 and PerR.

The third and fourth putative Cap0037 binding sites were in the promoter regions of two genes encoding potential transcriptional regulators of the MarR family for *CA_C3283* and of a CRO repressor-like DNA-binding protein for *CA_C3645*. The Cap0037 binding site was located downstream of the transcription start point (TSP) of *CA_C3283*, which is part of a three-gene operon (*CA_C3283-CA_C3281*) including two genes encoding a potential ABC transporter. All three genes were upregulated in the Cap0037::*int* mutant under acidogenesis and alcohologenesis conditions. The regulatory effects of Cac3283 on the transcription of the two genes encoding the ABC transporter and the role of this ABC transporter are still unknown. *CA_C3645* is expressed as a monocistronic operon, and the Cap0037 binding site was located downstream of the TSP. Its transcription was upregulated under acidogenesis conditions only in the *CA_P0037*::*int* mutant.

The last four putative Cap0037 binding sites were located in the promoter region of a three-gene operon (*CA_C0984-CA_C0986*) encoding membrane proteins of unknown function, a three-gene operon (*CA_C0460-CA_C0462*) involved in butanoate metabolism, a gene (*CA_C3231*) encoding a potential HAD (haloacid dehalogenase-like) type of phosphatase, and the *CA_C0091* gene (encoding ketol-acid reductoisomerase, involved in valine, leucine, and isoleucine biosynthesis). However, the transcription levels of all of these genes were poorly affected in the *CA_P0037*::*int* mutant (see Table S2 in the supplemental material).

### *CA_P0037* inactivation affects the expression of the Rex, Fur, and PerR regulons.

The global effects of *CA_P0037* inactivation on *C. acetobutylicum* metabolism are presented in the supplemental material, and we will concentrate here on how *CA_P0037* inactivation affects the expression of the Rex, Fur, and PerR regulons. The redox-sensing transcriptional repressor (Rex) has been found to modulate its DNA binding activity in response to the NADH/NAD^+^ ratio and plays a role in the alcohologenic shift of *C. acetobutylicum*. Rex binding sites were determined in the promoters of different operons, the *adhE2*, *ldhA*, *crt*, *thlA*, *asrT*, *ptb*, and *nadA* operons (10, 11). Those operons were upregulated in the Rex mutant*.* The *ldhA* gene was the most induced gene, followed by *adhE2* (11). In the *CA_P0037*::*int* mutant, Rex expression was only 1.4-fold higher in acidogenesis and was unchanged in alcohologenesis and solventogenesis. However, except for the *thlA* gene, all of the other genes of the Rex regulon, such as *adhE2*, *ldhA*, *crt-bcd-etfAB-hbd*, *nadABC*, and *ptb-buk*, were upregulated to various degrees (see Table S3 in the supplemental material). As there are no putative Cap0037 binding sites in the promoter regions of all the genes or operons shown to be controlled by Rex, it is tempting to speculate that the effect of *CA_P0037* inactivation might be indirect and occur through an increase in the NADH/NAD^+^ ratio. The fact that *thlA* was unchanged or slightly downregulated in the *CA_P0037*::*int* mutant but highly upregulated in the Rex mutant suggests a more complex regulatory mechanism of *thlA* expression.

In a *perR* mutant or after the wild-type strain is exposed to oxygen, the genes encoding proteins involved in oxygen and reactive oxygen species (ROS) detoxification, including the reverse rubrerythins (*rbr3A-rbr3B*), desulfoferrodoxin (*dfx*), glutaredoxin (*CA_C2777*), rubredoxin (*rd* and *CA_C2778*), NADH-dependent rubredoxin oxidoreductase (*nror*), the oxygen-reducing flavoproteins (*fprA1* and *fprA2*), and a flavodoxin (*CA_C2452*), were upregulated (5). The *CA_P0037*::*int* mutant behaved quite similarly to the *perR* mutant under acidogenic and alcohologenic conditions (see Table S4 in the supplemental material). As shown above, a putative Cap0037 binding site was located 7 bp upstream of the −35 promoter region of *CA_C2777-CA_C2778* encoding a glutaredoxin and a rubredoxin, respectively. However, no putative Cap0037 binding sites were identified in the promoter regions of all of the other genes or operons shown to be controlled by PerR, and it is probable that the effect of *CA_P0037* inactivation is indirect.

Interestingly, we also found a common regulatory effect of Cap0037 with the *fur* mutant. Table S4 in the supplemental material compares the *CA_P0037*::*int* mutant with the *fur* mutant and the wild-type strain under iron limiting conditions. One of the most critical similarities is the overexpression of genes involved in iron transport and metabolism. As discussed above, the ferrous transport system FeoA (encoded by *CA_C1029-CA_C1032*), which contains a putative Cap0037 box and a Fur box in the promoter, was strikingly overexpressed in the *CA_P0037*::*int* mutant, the Fur mutant, and in the iron-limited WT. The second putative ferrous iron uptake system FeoB (encoded by *CA_C0447-CA_C0448*) for Fe^2+^ and the ferrichrome-dependent system (encoded by *CA_C0788-CA_C0791*) were also highly upregulated in all cases, although less significantly in the iron-limited WT. A flavodoxin (*fld1*)-encoding gene (*CA_C0587*) was strikingly upregulated in the iron-limited WT, the *fur* mutant, and the *CA_P0037*::*int* mutant (Table S4). A Fur box was found in the promoter of *fld1* (*CA_C0587*), but no putative Cap0037 box was identified. It has been well documented that the ferredoxin concentration is reduced dramatically in clostridia under low iron conditions, *fld1* is induced, and flavodoxin replaces ferredoxin as a single electron carrier. However, in the *CA_P0037*::*int* mutant, the expression level of *fdx* (*CA_C0303*), encoding the main ferredoxin, was not reduced. In iron deficiency or in a *fur* mutant or *CA_P0037* inactivation, the levels of genes belonging to the *ribGBAH* operon, which is involved in riboflavin synthesis, are strongly induced. Riboflavin biosynthesis genes were previously shown to be upregulated only by butyrate stress and not by acetate or butanol stress (23). Riboflavin is a precursor of the flavin mononucleotide (FMN) and flavin adenine dinucleotide (FAD) coenzymes. The expression of the *rib* operon was shown to be regulated by an FMN-sensing riboswitch element (24) in *Bacillus subtilis*, but the regulatory mechanism in *C. acetobutylicum* has yet to be characterized.

To summarize, the *CA_P0037*::*int* mutant cells behave as if they are Fe^2+^ limited, although they are in excess iron conditions. However, the upregulated Fur regulon in the mutant is an indirect regulatory effect rather than reflecting a real shortage in the intracellular Fe^2+^ concentration, and except for the FeoA-encoding operon (*CA_C1029-CA_C1032*), no Cap0037 box was identified in the promoter regions of the upregulated genes.

### Conclusion.

Collectively, these results demonstrate that in the strict anaerobe *C. acetobutylicum*, Cap0037, beside controlling its own gene, directly or indirectly controls a large set of genes belonging to the Rex, PerR, and Fur regulons in addition to the *sol* operon and *adc* gene that encode genes directly involved in solvent formation. These controls drastically affect the primary metabolism of *C. acetobutylicum*. However, more experiments will be needed to understand the mechanism(s) behind the global regulation by Cap0037*.*

## MATERIALS AND METHODS

### Bacterial strains, plasmids, and culture media.

The bacterial strains, plasmids, and primers used in this study are listed in Table S1 in the supplemental material. *C. acetobutylicum* Δcac1502 Δupp and its derivative mutants were grown in clostridial growth medium (CGM) for routine manipulation and in synthetic medium (SM) for sporulation and the production of solvents. A reinforced clostridial agar (RCA) plate with antibiotic at a pH of 5.8 was used after electroporation, and a pH of 6.5 was used for the replication of transformants. The *Escherichia coli* strain was grown in Luria-Bertani (LB) medium at 37°C. Antibiotics were used at the following concentrations: erythromycin (Erm) and clarithromycin at 40 µg/ml and thiamphenicol (Tm) at 10 µg/ml for *C. acetobutylicum* and ampicillin at 50 µg/ml for *E. coli.*

### Plasmid construction.

The *CA_P0037*::*int* mutant was successfully generated by using pCUI-Cap0037 (189s). This plasmid is derived from the pCUI plasmid, which was developed by our lab (20). The intron in this vector is smaller than in the pMTL007C-E2 system (18) because it does not contain a marker gene. The pCUI-Cap0037 (189s) plasmid was transformed into *Clostridium acetobutylicum* Δcac1502 Δupp by electroporation without prior methylation (25), and the transformants were selected on an RCA plate at a pH of 6.5 containing thiamphenicol at 40 µg/ml. In order to select for mutants, screening by PCR with two external primers must be performed. In the plasmid pCUI-Cap0037 (189s), the retargeting region was designed via the Perutka algorithm for the position 189/190 sense strand. In the presence of the *ltrA* gene, the integrants could lose the intron due to mRNA maturation. As a result, after screening and selecting for insertion, the plasmid pCUI-Cap0037 (189s) in the mutant was cured by spreading the cells on RCA at a pH of 6.5 containing 5′-fluorouracil (5-FU) at 1 mM (25). The colonies were then checked for plasmid loss by testing their sensitivity to Tm, Erm, and PCR using LtrA 5D and LtrA 3R primers that are specific for the vector backbone. The mutant *CA_P0037*::*int* was successfully generated by this second strategy.

For the complementation experiment, *CA_P0037* CDS and its ribosomal binding site (RBS) were amplified by Cap0037-F-RBS BamHI and Cap0037-R-*Sfo*I and cloned into a pSOS95 shuttle vector (26) by two restriction sites, BamHI and SfoI, to obtain the plasmid pSOS95-Cap0037. This pSOS95 vector allows the expression of *CA_P0037* under the constitutive *thlA* promoter. The plasmid pSOS95-Cap0037 was then electroporated into the *CA_P0037*::*int* mutant and selected on an RCA plate with Erm at 40 µg/ml. The complementary strain carrying the plasmid was maintained in liquid SM culture under pressure from clarithromycin at 40 µg/ml.

Plasmid pBS2 was used for heterologous production of Cap0037. For construction of plasmid pBS2, the 630-bp *CA_P0037* open reading frame was amplified by PCR using primers ygaS1hinCl and yghSrückCl with *C. acetobutylicum* WT genomic DNA as the template. The PCR product was digested with NdeI and HindIII and ligated into digested vector pET29a(+). Vector pET29a(+) contains a His tag sequence that is added to the C terminus of the produced protein.

Plasmids pDrive_86kurz, pDrive_144, pDrive_*adc*Start, and pDrive_*sol* were used for sequencing reaction of DNase I protection assay (DNA footprinting). For construction of pDrive_86kurz, pDrive_144, pDrive_*adc*Start, and pDrive_*sol*, probes 85 (256 bp), 144 (249 bp), *adc*Start (263 bp), and *sol* (245 bp) were amplified by PCR using genomic DNA from *C. acetobutylicum* WT as the template and primers 86 fwd (fwd stands for forward) and 85 rev (rev stands for reverse), 144 rev and 145 fwd, adcstart FP EcoRI fwd and adcstart FP EcoRV rev, and sol FP EcoRI fwd and sol FP EcoRV rev, respectively. PCR products were ligated into pDrive cloning vector. The generated PCR probes, probes 85 (256 bp), 144 (249 bp), *adc*Start (263 bp), and *sol* (245 bp), which contain parts of respective promoter regions, were used as probes for DNase I protection assay (DNA footprinting).

### Continuous culture.

Continuous fermentation was performed as previously described (12). The cultures were maintained under acidogenesis, solventogenesis and alcohologenesis conditions, fed with 995 mM total carbon, and maintained at a dilution rate of 0.05 h^−1^. Samples for the production profile analysis and mRNA extraction were prepared under steady-state conditions. Results for the *Clostridium acetobutylicum* Δcac1502 Δupp Cap0037::*int* mutant were compared with the control strain *Clostridium acetobutylicum* Δcac1502 Δupp (12).

For the complementary strain, continuous culture under acidogenic conditions was performed in the presence of clarithromycin at 40 µg/ml for a short period to avoid losing the plasmid. The production profile was analyzed and compared with the *CA_P0037*::*int* mutant.

### Isolation of total mRNA and microarray.

Total RNA was isolated from continuous cultures using the RNeasy kit protocol (Qiagen, Hilden, Germany). The protocol and microarray slides used in this study were the same as described elsewhere (12) to obtain comparable results.

### Analytical methods.

The concentrations of the substrate and fermentation products were measured by high-pressure liquid chromatography (HPLC) (Agilent 1200 series, Massy, France). The separation was obtained with an Aminex HPX-87H column (300 by 7.8 mm) (Bio-Rad), at 14°C with 0.5 mM H_2_SO_4_ at a flow rate of 0.5 ml/min. Detection was achieved by reading the refractive index and UV absorbance (210 nm).

### Southern blot analysis.

Genomic DNA of the *CA_P0037*::*int* mutant was digested with EcoRI restriction enzyme. The probe for hybridization was prepared by PCR using Intron Probe-F (F stands for forward) and Intron Probe-R (R stands for reverse) primers and was then labeled using the DIG High Prime DNA labeling and detection starter kit II (Roche). The protocols for hybridization and detection were performed according to the Roche instructions.

### Expression and purification of His-tagged protein.

*E. coli* BL21(DE3) cells (Novagen R and D Systems) containing pBS2 was used for overproduction of protein Cap0037. The cells were grown aerobically at 37°C in LB medium supplemented with either kanamycin (50 µg/ml) or ampicillin (100 µg/ml) to an optical density at 600 nm (OD_600_) of 0.7 and induced by the addition of 1 mM isopropyl-β-d-1-thiogalactopyranoside (IPTG) at 37°C until stationary phase was reached. The cells were then harvested by centrifugation (4,300 × *g*, 10 min, 4°C) and washed twice with buffer containing imidazole (10 mM). The weight of the cell pellet (wet weight) was determined and solubilized in 3 volumes of the buffer mentioned above. The cells were disrupted using a French pressure cell (4 MPa) (French Pressure Cell/Press, G. Heinemann Ultraschall und Labortechnik, Schwäbisch Gmünd, Germany). The fusion protein was purified by nickel-nitrilotriacetic acid (NTA) affinity chromatography using Ni-NTA agarose (Qiagen GmbH, Hilden, Germany). For elution, imidazole buffers with 75, 100, 200, and 250 mM imidazole were used. The purity of the protein was analyzed by SDS-polyacrylamide gel electrophoresis, and protein concentration was quantified by Pierce BCA protein assay kit (Pierce, Rockford, IL, USA) according to the instructions provided by the manufacturer.

### Electrophoretic mobility shift assays (EMSAs).

For electrophoretic mobility shift assays (EMSAs) of the *adc*, *CA*_*P0037*/*CA*_*P0036*, and *sol* operon, the respective DNA probes were amplified by PCR using genomic DNA from *C. acetobutylicum* ATCC 824 as the template. For amplifying the 5′ untranslated region of the *adc* gene, the adc probe (318 bp), primers adc_forward and adc_reverse (see Table S1 in the supplemental material) were used. For EMSAs to the 5′ untranslated region of the *CA_P0037-CA_P0036* operon, the CAP0037 probe (539 bp), primers 94_fwd and PEX1_kurz were used. For EMSAs to the 5′ untranslated region of the *sol* operon, the sol probe (219 bp) and primers sol_frag_F and sol_frag_R were used. The adcint probe (233 bp) was used as a negative control and was amplified out of the internal region of the *adc* gene, using primers adcmit1 and adcmit2. In the binding assays, DNA probes were mixed with increasing amounts of the respective fusion proteins in 20-µl reaction mixtures with 0.5 µl poly(dI-dC) (2 µg/µl) and 2-µl band shift buffer (200 mM Tris, 500 mM Na glutamate, 100 mM MgCl_2_ ⋅ 6H_2_O, 63.6 mM EDTA, 0.5% Nonidet P-40 [vol/vol], 50% glycerol [vol/vol]). After incubation for 20 min at room temperature, samples were loaded on a 2% agarose gel in 1× TAE buffer (200 mM Tris, 100 mM acetate, 5 mM EDTA [pH 7.5]) and separated by electrophoresis (70 V, 50 min). The gel was stained using ethidium bromide.

For electrophoretic mobility shift assays of *ldhA* and *fld1*, PCR DNA fragments of *ldhA* and *fld1* promoters were generated by two pairs of primers (ldh-prom-F [prom stands for promoter] and ldh-prom-R primers and fld1-prom-F and fld1-prom-R primers) and DIG labeled (with DIG High Prime DNA labeling and detection starter kit II [Roche]). Binding assays were done as described above. The detection of DNA shifts was performed according to Roche protocol.

### DNase I protection assays (DNA footprinting).

To determine the exact binding sites of protein Cap0037 at the 5′ untranslated regions of the *CA_P0037-CA_P0036* operon, *adc* operon, and *sol* operon, different DNA fragments (probes) were amplified by PCR (see “Plasmid construction” above3.2). The primers generated restriction sites at both ends of the probes (see “Plasmid construction” above3.2). The probes were radioactively 5′ labeled using [γ-^32^P]ATP. Labeling was performed in a 25-µl reaction mixture with 20 pmol DNA, 2 µl polynucleotide kinase (10 U/µl) (Thermo Scientific, St. Leon-Rot, Germany), 2.5 µl reaction buffer (10× binding buffer [see below] and 2 µl bovine serum albumin [BSA] 100 mg/ml [wt/vol]), and 4 pmol [γ-^32^P]ATP (20 µCi). The reaction mixture was incubated at 37°C for 1 h. One end of the DNA was digested using EcoRV, removing a 5′-labeled phosphate. Purification of the labeled DNA was carried out by Microspin G-25 columns (GE Healthcare, Buckinghamshire, United Kingdom) according to the instructions of the manufacturer. For protein binding, the respective radiolabeled DNA probe was mixed with increasing amounts of Cap0037 in a 20-µl reaction mixture together with 0.5 µl poly(dI-DC) (2 µg/µl) and 2 µl of10× binding buffer (500 mM Tris, 500 mM KCl, 50 mM MgCl_2_ ⋅ 6H_2_O, 10 mM EDTA). After incubation for 30 min at 37°C, 1.1 µl buffer R1 (120 mM MgCl_2_ ⋅ 6H_2_O, 120 mM CaCl_2_ ⋅ 2H_2_O) and 0.1 U DNase (Thermo Scientific, St. Leon-Rot, Germany) were added. The reaction was stopped after just 1 min by adding 1 µl EDTA (0.5 M, pH 8) and placing the reaction mixture on ice. The sample was purified by phenol chloroform extraction, and the DNA was ethanol precipitated and suspended in 3 µl of sequencing gel loading buffer (30 mg bromophenol blue, 30 mg xylene cyanole, 200 mM EDTA [pH 8], and 10 ml formamide). After an incubation at 80°C for 2 min, the DNase I digestion product was loaded onto a 7 M urea−6% polyacrylamide gel and separated at 1,500 V, 46 mA, and 60 W for 2 h. The dideoxy sequencing reaction was performed using the respective plasmid (see “Plasmid construction” above3.2) and T7 sequencing kit (Affymetrix, Santa Clara, CA, USA), and run in the same gel as the footprint sample. The gel was vacuum dried, exposed on Hyperfilm MP (Amersham Biosciences, Europe GmbH, Freiburg, Germany) for 24 h and developed with VCURIX 60V (AGFA Graphics GmbH & Co. KG, Düsseldorf, Germany).

### Bioinformatic tools. (i) BLAST searches, amino acid sequence alignments, and phylogenetic analysis.

Identification of orthologs was performed using the BLAST tool provided by NCBI (27). Amino acid sequence alignment and the phylogenetic tree were processed using the phylogeny program (28).

### (ii) Transmembrane protein prediction.

Cap0036 amino acid sequence was used as an input to find possible transmembrane regions using two bioinformatic web-based tools, TMpred (15) and OCTOPUS (16).

### (iii) DNA binding motif search and promoter element prediction.

Amino acid sequences of Cap0037 were subjected to a search for the helix-turn-helix DNA binding motif using the NPS@ web-based biotool (https://npsa-prabi.ibcp.fr/) (17). To predict the promoters of different genes, BPROM web-based tool was used (21).

### (iv) Putative Cap0037 regulon.

Construction of putative Cap0037 regulon was performed using an established comparative genomics method (29) implemented in the RegPredict webserver (http://regpredict.lbl.gov) (30) and Artemis Java Webstart (31). Genes with potential upstream binding sites that had high scores and/or were conserved were rebuilt to improve search accuracy. Genome sequences and annotations of *C. acetobutylicum* were obtained from GenBank (http://www.ncbi.nlm.nih.gov/GenBank/).

### Western blot analysis.

Aliquots containing 100 µg of purified His-tagged Cap0037 were mixed with an equal amount of adjuvant (Specoll) and injected into a New Zealand White rabbit. Antiserum was collected 9 weeks after the first immunization. Western blot analyses were carried out with antiserum diluted 1,000-fold by using a standard protocol (32).

### Microarray data accession number.

The microarray data can be accessed at GEO through accession numbers GSE81273 and GSE69973 for the *CA_P0037*::*int* mutant and the control strain (12), respectively.

## SUPPLEMENTAL MATERIAL

Data Set S1  Transcriptomic and proteomic data Download Data Set S1, XLSX file, 2.3 MB

Figure S1  Phylogenetic trees of Cap0036 (A) and Cap0037 (B) sequences from *Clostridium acetobutylicum* and their neighbor proteins from other bacteria. The neighbor proteins were selected by running BLAST on the NCBI database. Cap0036 and Cap0037 proteins from *C. acetobutylicum* are shown in blue. Download Figure S1, DOC file, 0.1 MB

Figure S2  Metabolic fluxes of mutant *CA_P0037*::*int* versus control strain in different metabolic states, such as alcohologenesis (AL) and solventogenesis (SO). (A and B) Carbon fluxes; (C and D) electron fluxes. All values are normalized to the flux of the initial carbon source (millimoles per gram [dry cell weight] per hour). Glucose flux is normalized and set at 100 for acidogenesis and solventogenesis, and the sum of glucose and half of the glycerol normalized as 100 for alcohologenesis. The values of the corresponding mutant are shown in blue, and those of the control strain are shown in green. The control data were from reference 12. Download Figure S2, DOC file, 0.3 MB

Figure S3  (A) EMSAs using the promoter region of *adc* and the Cap0037 protein. Lanes 1 to 8, 0, 0.2, 0.3, 0.5, 0.6, 0.7, 0.8, and 0.9 µg protein, respectively; lanes 9 to 12, 0, 0.2, 0.6, and 0.9 µg protein, respectively. (B) EMSAs using the promoter region of the *sol* operon and the Cap0037 protein. Lanes 1 to 5, 0, 0.28, 0.42, 0.7, and 1 µg protein, respectively; lanes 6 to 8, 0, 0.42, and 1 µg protein, respectively. Download Figure S3, DOC file, 0.8 MB

Figure S4  (A) DNase I protection assay I (DNA footprinting) of Cap0037 interacting with the *adc* promoter region (probe 144). End-labeled DNA fragment carrying the promoter region of the *adc* operon was incubated with different concentrations of Cap0037, subjected to DNase I cleavage, and analyzed on a sequencing gel. The sequencing reaction was performed with plasmid pDrive_144. (B) DNA footprinting of Cap0037 interacting with the *adc* promoter region (transcription start) (probe *adc*Start). End-labeled DNA fragment carrying the promoter region of the *adc* operon (transcription start) was incubated with different concentrations of Cap0037, subjected to DNase I cleavage, and analyzed on sequencing gels. The sequencing reaction was performed with plasmid pDrive_*adc*Start. (C) DNA footprinting of Cap0037 interacting with the *sol* promoter region (probe *sol*). End-labeled DNA fragment carrying the promoter region of the *sol* operon was incubated with different concentrations of Cap0037, subjected to DNase I cleavage, and analyzed on sequencing gels. The sequencing reaction was performed with plasmid pDrive_*sol*. Lanes 1 to 7 contain 0 to 2,308 nM Cap0037. The assigned region on the right side indicates the region protected by Cap0037 and the respective sequence. Download Figure S4, DOC file, 0.4 MB

Table S1  Bacterial strains, plasmids, and primers used in this study Table S1, DOCX file, 0.03 MB

Table S2  Prediction of the putative Cap0037 regulon and the corresponding relative transcript levels of those genes of the *CA_P0037*::*int* mutant in the three metabolic states, acidogenesis (AC), alcohologenesis (AL), and solventogenesis (SO). Orange letters are mismatched nucleotides compared to the binding box ATATTTTCATATAAAT in the *CA*_*P0037*/*CA*_*P0036* promoter. Table S2, DOCX file, 0.02 MB

Table S3  Relative transcript levels of genes belonging to the Rex regulon of the *C. acetobutylicum* CA_P0037::*int* mutant in the three metabolic states, acidogenesis (AC), alcohologenesis (AL), and solventogenesis (SO), and of the *rexA* mutant (data from reference 11). n.a., not available; n.d., not detected. Table S3, DOCX file, 0.02 MB

Table S4  Relative transcript levels of selected genes of the *CA_P0037*::*int* mutant in the three metabolic states (acidogenesis [AC], alcohologenesis [AL], and solventogenesis [SO]). The transcript levels in the oxygen-exposed WT (+O_2_ WT), Δ*perR* mutant, iron-limited WT (−Fe WT), and *fur*::*int* mutant are shown. Data for the oxygen-exposed WT and the Δ*perR* mutant are from reference 5, and data for iron-limited WT and *fur*::*int* mutant are from reference 6. n.a., not available. Table S4, DOCX file, 0.02 MB
