# Supplementary material for: Cap0037, a Novel Global Regulator of Clostridium acetobutylicum Metabolism
Source: mBio. 2016 Oct 4;7(5):e01218-16. doi: 10.1128/mBio.01218-16 (PMC5050335; doi:10.1128/mBio.01218-16)
Supplement: Figure S1 — Phylogenetic trees of Cap0036 (A) and Cap0037 (B) sequences from Clostridium acetobutylicum and their neighbor proteins from other bacteria. The neighbor proteins were selected by running BLAST on the NCBI database. Cap0036 and Cap0037 proteins from C. acetobutylicum are shown in blue. Download [file mbo005162999sf1.doc]

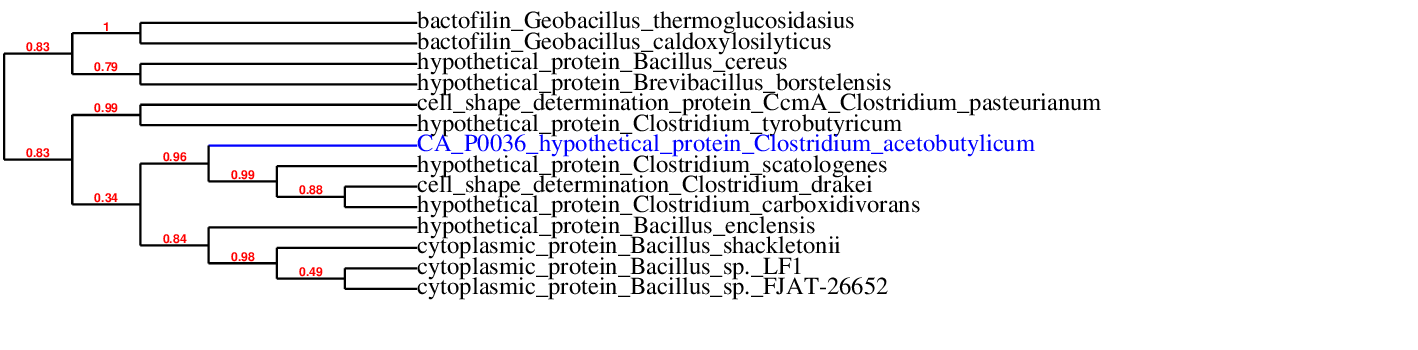

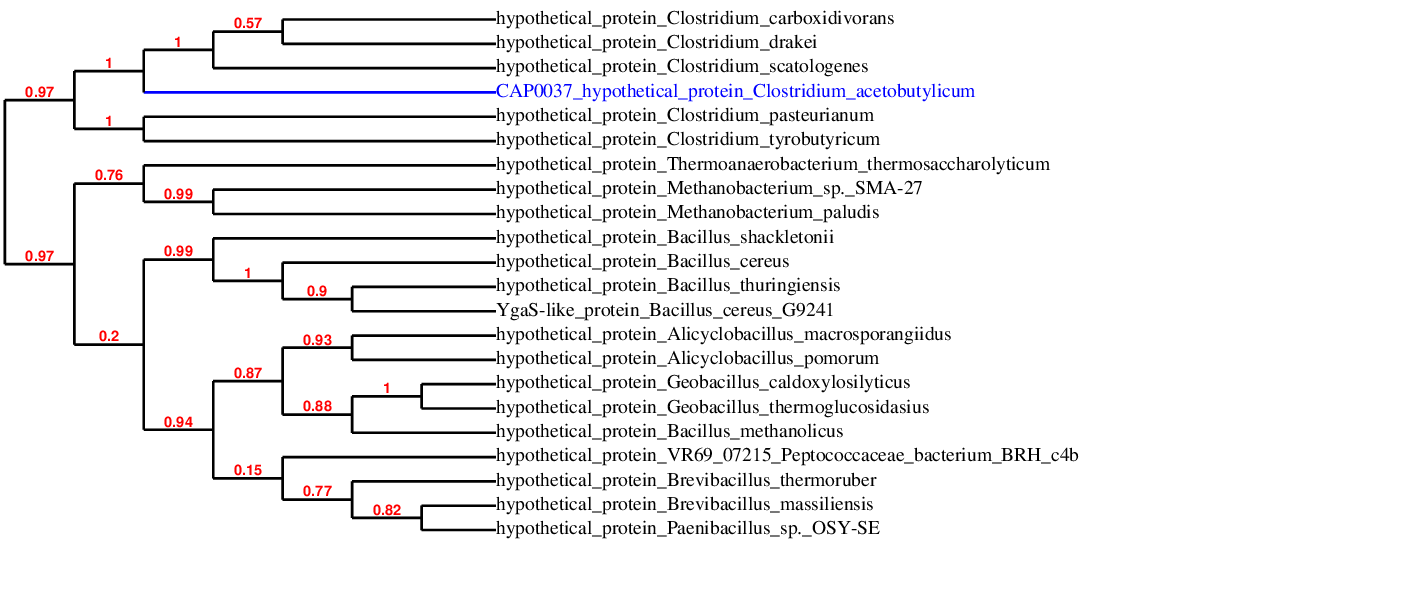


**A**

**B**

**Figure S- Phylogenetic trees of Cap0036 (A), Cap0037 (B) sequences from *Clostridium acetobutylicum* and their neighbor proteins from other bacteria**. The neighbor proteins were selected by running BLAST on NCBI database. Cap0036 and Cap0037 proteins from *C. acetobutylicum* are shown in blue.
