# Supplementary material for: Cap0037, a Novel Global Regulator of Clostridium acetobutylicum Metabolism
Source: mBio. 2016 Oct 4;7(5):e01218-16. doi: 10.1128/mBio.01218-16 (PMC5050335; doi:10.1128/mBio.01218-16)
Supplement: Figure S2 — Metabolic fluxes of mutant CA_P0037::int versus control strain in different metabolic states, such as alcohologenesis (AL) and solventogenesis (SO). (A and B) Carbon fluxes; (C and D) electron fluxes. All values are normalized to the flux of the initial carbon source (millimoles per gram [dry cell weight] per hour). Glucose flux is normalized and set at 100 for acidogenesis and solventogenesis, and the sum of glucose and half of the glycerol normalized as 100 for alcohologenesis. The values of the corresponding mutant are shown in blue, and those of the control strain are shown in green. The control data were from reference 12. Download [file mbo005162999sf2.doc]

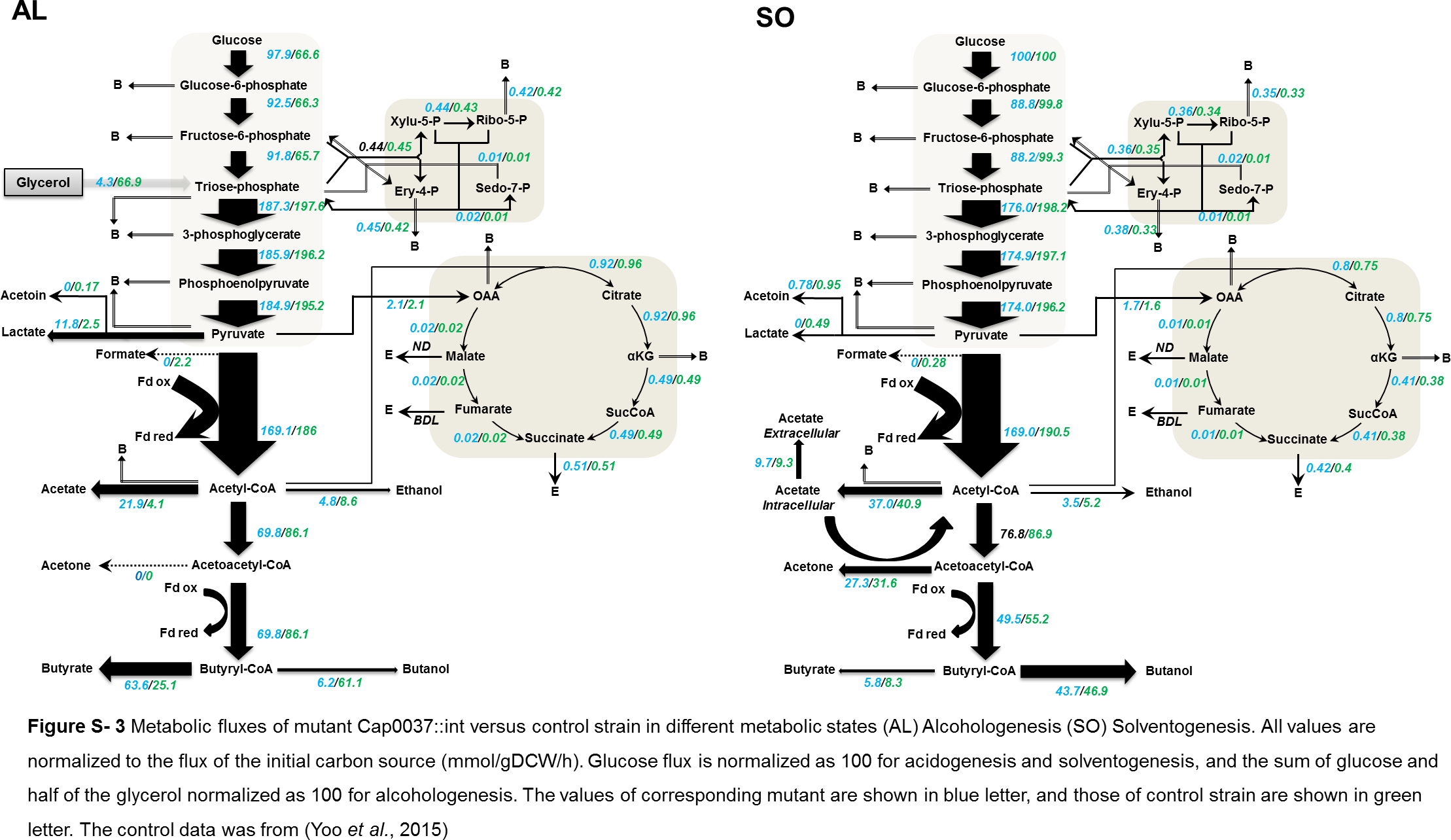

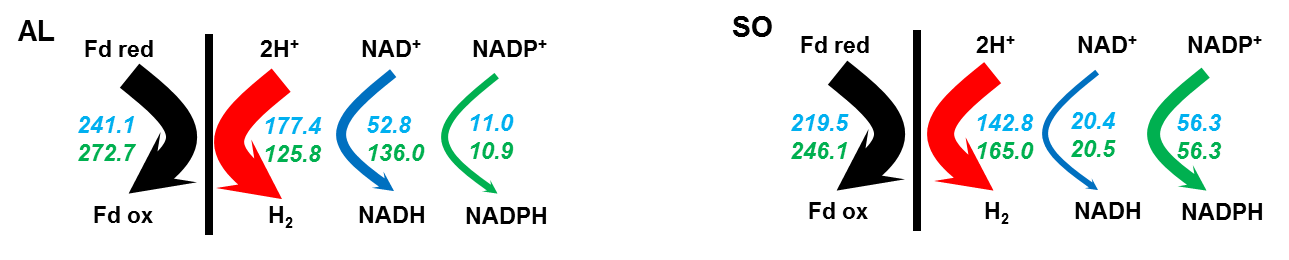


**(A)**

**(B)**

**(C)**

**(D)**

**Figure S- 2 Metabolic fluxes of mutant *CA_P0037::int* versus control strain in different metabolic states (AL) Alcohologenesis (SO) Solventogenesis**: Carbon fluxes **(A)** and **(B)** and Electron fluxes **(C)** and **(D)**. All values are normalized to the flux of the initial carbon source (mmol/gDCW/h). Glucose flux is normalized as 100 for acidogenesis and solventogenesis, and the sum of glucose and half of the glycerol normalized as 100 for alcohologenesis. The values of corresponding mutant are shown in blue letter, and those of control strain are shown in green letter. The control data was from (12)
