# Supplementary material for: Cap0037, a Novel Global Regulator of Clostridium acetobutylicum Metabolism
Source: mBio. 2016 Oct 4;7(5):e01218-16. doi: 10.1128/mBio.01218-16 (PMC5050335; doi:10.1128/mBio.01218-16)
Supplement: Figure S3 — (A) EMSAs using the promoter region of adc and the Cap0037 protein. Lanes 1 to 8, 0, 0.2, 0.3, 0.5, 0.6, 0.7, 0.8, and 0.9 µg protein, respectively; lanes 9 to 12, 0, 0.2, 0.6, and 0.9 µg protein, respectively. (B) EMSAs using the promoter region of the sol operon and the Cap0037 protein. Lanes 1 to 5, 0, 0.28, 0.42, 0.7, and 1 µg protein, respectively; lanes 6 to 8, 0, 0.42, and 1 µg protein, respectively. Download [file mbo005162999sf3.doc]

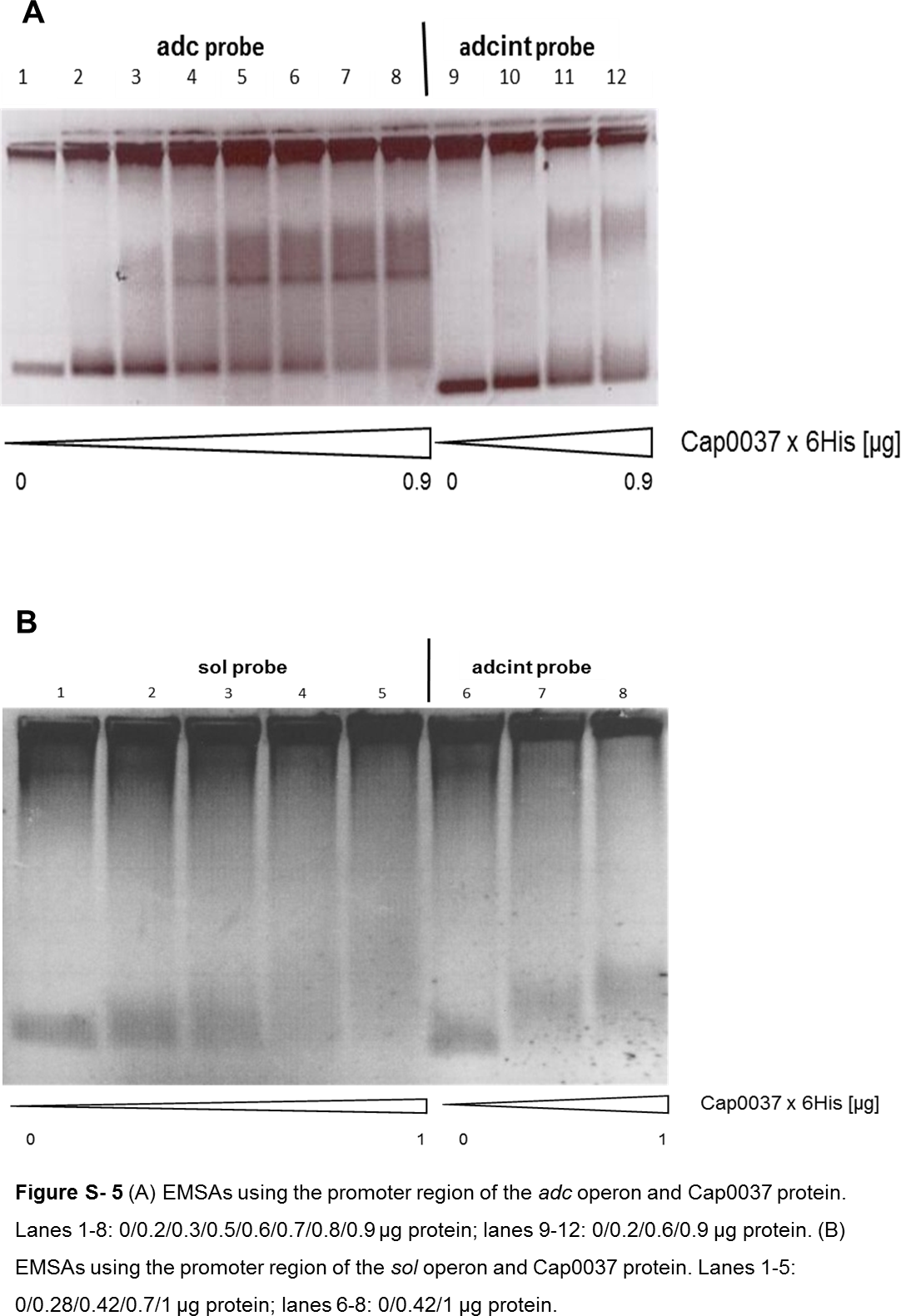


**Figure S- 3 (A)** EMSAs using the promoter region of *adc* and the Cap0037 protein. Lanes 1-8: 0/0.2/0.3/0.5/0.6/0.7/0.8/0.9 µg protein; lanes 9-12: 0/0.2/0.6/0.9 µg protein. **(B)** EMSAs using the promoter region of the *sol* operon and the Cap0037 protein. Lanes 1-5: 0/0.28/0.42/0.7/1 µg protein; lanes 6-8: 0/0.42/1 µg protein.
