# Supplementary material for: Cap0037, a Novel Global Regulator of Clostridium acetobutylicum Metabolism
Source: mBio. 2016 Oct 4;7(5):e01218-16. doi: 10.1128/mBio.01218-16 (PMC5050335; doi:10.1128/mBio.01218-16)
Supplement: Figure S4 — (A) DNase I protection assay I (DNA footprinting) of Cap0037 interacting with the adc promoter region (probe 144). End-labeled DNA fragment carrying the promoter region of the adc operon was incubated with different concentrations of Cap0037, subjected to DNase I cleavage, and analyzed on a sequencing gel. The sequencing reaction was performed with plasmid pDrive_144. (B) DNA footprinting of Cap0037 interacting with the adc promoter region (transcription start) (probe adcStart). End-labeled DNA fragment carrying the promoter region of the adc operon (transcription start) was incubated with different concentrations of Cap0037, subjected to DNase I cleavage, and analyzed on sequencing gels. The sequencing reaction was performed with plasmid pDrive_adcStart. (C) DNA footprinting of Cap0037 interacting with the sol promoter region (probe sol). End-labeled DNA fragment carrying the promoter region of the sol operon was incubated with different concentrations of Cap0037, subjected to DNase I cleavage, and analyzed on sequencing gels. The sequencing reaction was performed with plasmid pDrive_sol. Lanes 1 to 7 contain 0 to 2,308 nM Cap0037. The assigned region on the right side indicates the region protected by Cap0037 and the respective sequence. Download [file mbo005162999sf4.doc]

**1 2 3 4 5 6 7 A T G C**

**0 2038 nM**


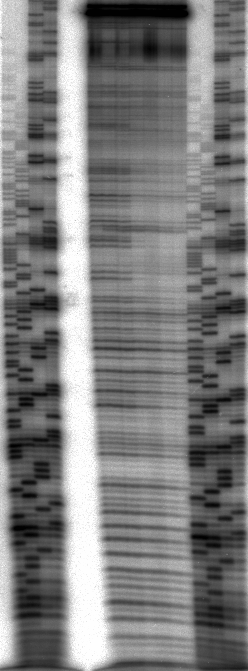


**ATAAGTTTATATAAAT**

**A**

**A T G C 1 2 3 4 5 6 7 A T G C**

**0 2038 nM**


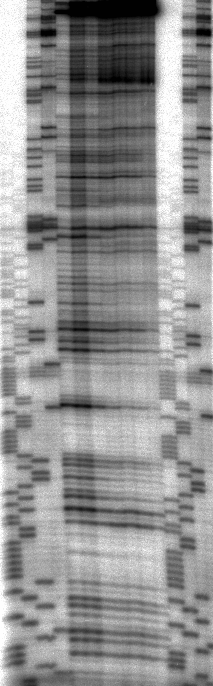


**TAATGTAAATATAAAT**

**B**

**0 2038 nM**

**A T G C 1 2 3 4 5 6 7 A T G C**


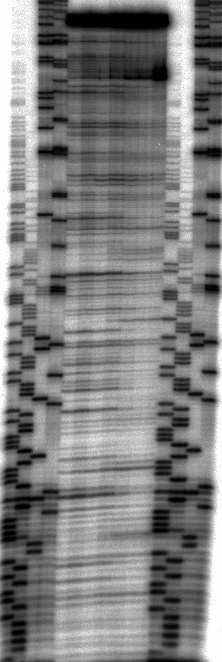


**TAAATATACTGATAAT**

**C**

**Figure S- 4 (A)** DNAase I protection assay I (DNA Footprinting) of Cap0037 interacting with *adc* promoter region (probe 144). End-labeled DNA fragment carrying the promoter region of the adc operon was incubated with different concentrations of Cap0037, subjected to DNAase I cleavage, and analyzed on sequencing gel. Sequencing reaction was performed with plasmid pDrive_144.

**(B)** DNA footprinting of Cap0037 interacting with *adc* promoter region (transcription start) (probe *adc*Start). End-labeled DNA fragment carrying the promoter region of the *adc* operon (transcription start) was incubated with different concentration of Cap0037, subjected to DNAase I cleavage, and analyzed on sequencing gels. Sequencing reaction was performed with plasmid pDrive_*adc*Start.

**(C)** DNA Footprinting of Cap0037 interacting with *sol* promoter region (probe *sol*). End-labeled DNA fragment carrying the promoter region of the *sol* operon was incubated with different concentration of Cap0037, subjected to DNAase I cleavage, and analyzed on sequencing gels. Sequencing reaction was performed with plasmid pDrive_*sol*. Line 1-7: 0-2308 nM Cap0037. The assigned region on the right side indicates the region protected by Cap0037 and the respective sequence.
