# Supplementary material for: Cap0037, a Novel Global Regulator of Clostridium acetobutylicum Metabolism
Source: mBio. 2016 Oct 4;7(5):e01218-16. doi: 10.1128/mBio.01218-16 (PMC5050335; doi:10.1128/mBio.01218-16)
Supplement: Table S1 — Bacterial strains, plasmids, and primers used in this study [file mbo005162999st1.docx]

**Table S- 1** Bacterial strains, plasmids and primers used in this study

| **Strain/plasmid** | **Relevant characteristics** | **Source** |
| --- | --- | --- |
|  | ***Clostridium acetobutylicum* and *Escherichia coli* strains** | |
| Control strain | *Clostridium acetobutylicum* *∆cac1502∆upp* | (1) |
| Mutant Cap37::int | *Clostridium acetobutylicum* *∆cac1502∆uppcap0037::int(189s)* | This study |
| Complementary strain | *Clostridium acetobutylicum* *∆cac1502∆uppcap0037::int(189s) pSOS95-cap0037* | This study |
| *Escherichia coli* TOP10 | *F- mcrA Δ(mrr-hsdRMS-mcrBC) φ80lacZΔM15 ΔlacX74 nupG recA1 araD139 Δ(ara-leu)7697 galE15 galK16 rpsL(Str^R^) endA1 λ^-^* | Invitrogen |
|  | **Plasmids** |  |
| pCR4-TOPO-Blunt | Ap^r^ Km^r^ | Invitrogen |
| pMTL007C-E2 | Cm^R^, Clostridial expression vector for the expression of ClosTron containing ErmRAM | (2) |
| pMTL007C-E2::cap37(277a) | Cm^R^, Clostridial expression vector for the expression of ClosTron containing ErmRAM, retargeting *CA_P0037* 277\|278a | This study |
| pCUI | *catP, erm, ltrA, repL, upp*, ColE1 origin, intron | (3) |
| pCUI-cap37(189s) | *catP, erm, ltrA, repL, upp*, ColE1 origin, intron retargeting *CA_P0037* 189\|190s | This study |
| pSOS95 | Ap^r^ MLS^r^, *repL* gene, ColE1 origin | (4) |
| pSOS95-Cap0037 | Ap^r^ MLS^r^, *repL* gene, ColE1 origin, Cap0037 operon | This study |
| pBS2 | pET29a(+), adcRx6His-Fusion | This study |
| pDrive | Ap^R^, Km^R^, pMB1 origin, *lac*POZˈ | This study |
| pDrive_adcR | pDrive vector with the promoter region of *CA_P0037/CA_P0036* (+487 to -108) | This study |
| pDrive_86kurz | pDrive vector with the promoter region of *CA_P0037/CA_P0036* (-219 to +21*)* | This study |
| pDrive_144 | pDrive vector with the promoter region *of CA_P0037/CA_P0036* (-233 to +6) | This study |
| pDrive_*adc*Start | pDrive vector with the promoter region of the *adc* operon (-105 to +49) | This study |
| pDrive_*sol* | pDrive vector with the promoter region of the *sol* operon (-233 to -15) | This study |

| **Primer** | **Sequence** |
| --- | --- |
| CA_P0037n-189\|190s -IBS | aaaaaagcttataattatccttagaaaacattaaggtgcgcccagatagggtg |
| CA_P0037n-189\|190s -EBS1d | cagattgtacaaatgtggtgataacagataagtcattaagagtaacttacctttctttgt |
| CA_P0037n-189\|190s -EBS2 | tgaacgcaagtttctaatttcgattttttctcgatagaggaaagtgtct |
| EBS universal | cgaaattagaaacttgcgttcagtaaac |
| CAP0037-277\|278a-IBS | aaaaaagcttataattatccttatgtttcgttccagtgcgcccagatagggtg |
| CAP0037-277\|278a-EBS1d | cagattgtacaaatgtggtgataacagataagtcgttccagctaacttacctttctttgt |
| CAP0037-277\|278a-EBS2 | tgaacgcaagtttctaatttcggttaaacatcgatagaggaaagtgtct |
| CAP0037-R-b | ttctctagcgtttacttttagaacaagag |
| CAP0037-F-b | atggatgaaactaatttaatttctaaaa |
| CAP0037_F | attttccccaaatctttcagagctttc |
| CAP0037_R | taagttcattatagtttgcgaaacaatg |
| cap37-F-RBS-BamH1 | aaaaaaggatccaggatctcaagcaaatcgcagaggacacagggtatggatgaaactaatttaatttctaaaaaagaacttc |
| cap37-R-SfoI | ttttttttggcgccttatagtgataattttgattttaattcttctaaacattctc |
| Intron probe-F | accctcttcgggaaccgtacg |
| Intron probe-R | gacttaacactaactggggataccc |
| REP-upp-F | aaaacagctgggaggaatgaaataatgagtaaagttacac |
| REP-upp-R | aaaacagctgttattttgtaccgaataatctatctccagc |
| ygas1hincl | gaatagccatatggatgaaac |
| ygas2rückcl | tcctttaagctttagtgataattttgattttaattcttctaaac |
| PEX1_kurz | ggcaagctcatctaatgatacatccc |
| PEX2_lang | cccggattttaggaacctatctaatatagtc |
| adcRpDrive | cactccttatctatagttcg |
| adcRpDrive_rev | ctacgtcacaatatcggtcatc |
| 86 fwd | gaattccctccaatattatc |
| 85 rev | gatatccctccagttattctc |
| 144 rev | agagaattcccattttaccttctattctaac |
| 145 fwd | agagatatccattataacacatattg |
| adcstart FP EcoRV fwd | acagatatcggcgaagttaatggcgtg |
| adcstart FP EcoRI rev | acagaattcgcagaatttttaggaagtg |
| sol FP EcoRI fwd | acagaattcctcaattctatttatgctcc |
| sol FP EcoRV rev | acagatatccaataaaaagcataggtc |
| adc_forward | gccaacattgcctataag |
| adc_reverse | gttaatggcgtgctaatttg |
| 94_fwd | ctttatcaacttcctcaacggg |
| adcmit1 | gatgagcccttagtcagg |
| adcmit2 | cctactaaagtatctgaatcc |
| sol_frag_F | actcaattctatttatgctcc |
| Sol_frag_R | gcataggtcaaaaatataacag |
| ldh-prom-F | ctgcataatataaaatgcagatttttttaaac |
| ldh-prom-R | acaaatcctgctccgattactg |
| fld1-prom-F | gttatgtagaatttatatacataattagagc |
| fld1-prom-R | caataaattatgtt tattttcacc |

1. **Yoo M**, **Bestel-Corre G**, **Croux C**, **Riviere A**, **Meynial-Salles I**, **Soucaille P**. 2015. A Quantitative System-Scale Characterization of the Metabolism of *Clostridium acetobutylicum*. MBio **6**:e01808–15.

2. **Heap JT**, **Kuehne SA**, **Ehsaan M**, **Cartman ST**, **Cooksley CM**, **Scott JC**, **Minton NP**. 2010. The ClosTron: Mutagenesis in Clostridium refined and streamlined. J Microbiol Methods **80**:49–55.

3. **Soucaille P**. December 2010. Process for the stable gene interruption in clostridia. U.S. Provisional Application No. 61/220,606.

4. **Raynaud C**, **Sarçabal P**, **Meynial-Salles I**, **Croux C**, **Soucaille P**. 2003. Molecular characterization of the 1,3-propanediol (1,3-PD) operon of *Clostridium butyricum*. Proc Natl Acad Sci U S A **100**:5010–5.
