# Supplementary material for: Cap0037, a Novel Global Regulator of Clostridium acetobutylicum Metabolism
Source: mBio. 2016 Oct 4;7(5):e01218-16. doi: 10.1128/mBio.01218-16 (PMC5050335; doi:10.1128/mBio.01218-16)
Supplement: Table S2 — Prediction of the putative Cap0037 regulon and the corresponding relative transcript levels of those genes of the CA_P0037::int mutant in the three metabolic states, acidogenesis (AC), alcohologenesis (AL), and solventogenesis (SO). Orange letters are mismatched nucleotides compared to the binding box ATATTTTCATATAAAT in the CA_P0037/CA_P0036 promoter. [file mbo005162999st2.docx]

**Table S- 2** Prediction of putative Cap0037 regulon and the correspondent relative transcript levels of those genes of *CA_P0037::int* mutant in the three metabolic states: acidogenesis (AC), alcohologenesis (AL), and solventogenesis (SO). Letters in orange are mismatched nucleotides compared to the binding box *ATATTTTCATATAAAT* in Cap0037 promoter.

| **ORF** | **Sequences** | **CDS** | **Function** | **Fold regulation** | | |
| --- | --- | --- | --- | --- | --- | --- |
|  |  |  |  | **AC** | **AL** | **SO** |
| CAP0037/0036 | ***ATATTTTCATATAAAT*** | CAP0037 | Uncharacterized protein | 0.962 | 50.716 | 68.558 |
|  |  | CAP0036 | Uncharacterized protein | 1.116 | 45.455 | 59.483 |
| CAC3283/3282/3281 | ***ATATTTTTATATAAAT*** | CAC3283 | Transcriptional regulator, MarR/EmrR family | 2.515 | 2.151 | 1.223 |
|  |  | CAC3282 | ABC-type multidrug/protein/lipid transport system, ATPase component | 4.146 | 2.427 | 1.858 |
|  |  | CAC3281 | ABC-type multidrug/protein/lipid transport system, ATPase component | 3.030 | 1.759 | 1.054 |
| CAC3231 | ***AGATTTTAATATAAAT*** | CAC3231 | Predicted phosphatase, HAD superfamily | 1.515 | 1.419 | 1.285 |
| CAC2777/2778 | ***ATATTTTTATTTAAAT*** | CAC2777 | Glutaredoxin | 7.201 | 3.447 | 1.623 |
|  |  | CAC2778 | Rubredoxin | 8.474 | 4.304 | 2.002 |
| CAC0091 | ***GTTTTTTTATATAAAT*** | CAC0091 | Ketol-acid reductoisomerase | 1.157 | 0.584 | 0.746 |
| CAC0460/0461/0462 | ***ATATATAAATATAAAT*** | CAC0460 | CBS-domain containing protein, YHDP *B.subtilis* ortholog | 1.758 | 1.066 | 1.280 |
|  |  | CAC0461 | Mercuric resistance operon regulatory protein, MerR family | 1.562 | 1.177 | 1.047 |
|  |  | CAC0462 | Trans-2-enoyl-CoA reductase | 1.677 | 1.085 | 1.300 |
| CAP0165 | ***ATAAGTTTATATAAAT*** | CAP0165 | Acetoacetate decarboxylase | 0.150 | 0.397 | 0.382 |
| CAC0984/0985/0986 | ***ATATTGACATTTAAAT*** | CAC0984 | ABC transporter, ATP-binding protein | 1.098 | 0.559 | 2.064 |
|  |  | CAC0985 | ABC transporter, permease component | 0.802 | 0.452 | 2.207 |
|  |  | CAC0986 | Lipoprotein, attached to the cytoplasmic membrane, NLPA family | 0.976 | 0.714 | 2.681 |
| CAC1029/1030/1031/1032 | ***ATATATACATTTAAAT*** | CAC1029 | FeoA-like protein, involved in iron transport | 195.62 | 243.32 | 76.236 |
|  |  | CAC1030 | FeoA-like protein, involved in iron transport | 252.12 | 283.60 | 83.150 |
|  |  | CAC1031 | FeoB-like GTPase, responsible for iron uptake | 220.99 | 337.12 | 68.679 |
|  |  | CAC1032 | Predicted transcriptional regulator | 318.15 | 452.71 | 82.493 |
| CAC3645 | ***ATTTTTTCTTTTAAAT*** | CAC3645 | CRO repressor-like DNA-binding protein | 2.422 | 0.702 | 0.798 |
