# Supplementary material for: Cap0037, a Novel Global Regulator of Clostridium acetobutylicum Metabolism
Source: mBio. 2016 Oct 4;7(5):e01218-16. doi: 10.1128/mBio.01218-16 (PMC5050335; doi:10.1128/mBio.01218-16)
Supplement: Table S3 — Relative transcript levels of genes belonging to the Rex regulon of the C. acetobutylicum CA_P0037::int mutant in the three metabolic states, acidogenesis (AC), alcohologenesis (AL), and solventogenesis (SO), and of the rexA mutant (data from reference 11). n.a., not available; n.d., not detected. [file mbo005162999st3.docx]

**Table S- 3** Relative transcript levels of genes belonging to Rex regulon of *CA_P0037::int* *C. acetobutylicum* mutant in the three metabolic states: acidogenesis (AC), alcohologenesis (AL) and solventogenesis (SO) and of Rex mutant (data published by (11)); n.a.: not available; n.d.: not detected)

| **ORF** | **Function** | **Fold of regulation** | | | |
| --- | --- | --- | --- | --- | --- |
|  |  | **Cap0037 ::int MT** | | | **Rex MT** |
|  |  | **AC** | **AL** | **SO** |  |
| CAC0267 | L-lactate dehydrogenase | 83.37 | 31.58 | 3.54 | 13.75 |
| CAC1023 | Nicotinate-nucleotide pyrophosphorylase | 2.62 | 0.84 | 0.34 | 1.79 |
| CAC1024 | Aspartate oxidase | 2.25 | 0.86 | 0.63 | 1.79 |
| CAC1025 | Quinolinate synthase | 2.33 | 0.93 | 0.54 | 2.53 |
| CAC1512 | Formate/nitrite family of transporter | n.d. | n.d. | n.d. | 2.04 |
| CAC1513 | Anaerobic sulfite reductase (Fe-S subunit) | n.d. | n.d. | n.d. | 2.41 |
| CAC1514 | Anaerobic sulfite reductase, B subunit | 0.61 | 0.72 | 0.77 | 2.67 |
| CAC1515 | Anaerobic sulfite reduction protein C, reductase | n.d. | n.d. | n.d. | 1.69 |
| CAC2708 | β-hydroxybutyryl-CoA dehydrogenase | 1.86 | 0.32 | 0.53 | 2.79 |
| CAC2709 | Electron transfer flavoprotein alpha-subunit | 2.60 | 0.51 | 0.75 | 1.55 |
| CAC2710 | Electron transfer flavoprotein beta-subunit | 2.02 | 0.38 | 0.68 | 2.17 |
| CAC2711 | Butyryl-CoA dehydrogenase | 1.46 | 0.96 | 0.83 | 2.81 |
| CAC2712 | Crotonase (3-hydroxybutyryl-COA dehydratase) | 1.85 | 0.94 | 1.04 | 2.43 |
| CAC2873 | Acetyl-CoA acetyltransferase | 0.98 | 0.25 | 0.43 | 12.46 |
| CAC3075 | Butyrate kinase, BUK | 0.89 | 0.65 | 0.71 | 1.97 |
| CAC3076 | Phosphate butyryltransferase | 1.41 | 0.79 | 0.86 | 6.07 |
| CAP0035 | Aldehyde-alcohol dehydrogenase, ADHE2 | 49.81 | 0.11 | 4.47 | 164.75 |
| CAC2713 | AT-rich DNA-binding protein | 1.44 | 0.79 | 0.92 | n.a. |

References

1. **Zhang L**, **Nie X**, **Ravcheev DA**, **Rodionov DA**, **Sheng J**, **Gu Y**, **Yang S**, **Jiang W**, **Yang C**. 2014. Redox-responsive repressor Rex modulates alcohol production and oxidative stress tolerance in *Clostridium acetobutylicum*. J Bacteriol **196**:3949–63.
