# Supplementary material for: Cap0037, a Novel Global Regulator of Clostridium acetobutylicum Metabolism
Source: mBio. 2016 Oct 4;7(5):e01218-16. doi: 10.1128/mBio.01218-16 (PMC5050335; doi:10.1128/mBio.01218-16)
Supplement: Table S4 — Relative transcript levels of selected genes of the CA_P0037::int mutant in the three metabolic states (acidogenesis [AC], alcohologenesis [AL], and solventogenesis [SO]). The transcript levels in the oxygen-exposed WT (+O2 WT), ΔperR mutant, iron-limited WT (−Fe WT), and fur::int mutant are shown. Data for the oxygen-exposed WT and the ΔperR mutant are from reference 5, and data for iron-limited WT and fur::int mutant are from reference 6. n.a., not available. [file mbo005162999st4.docx]

**Table S- 4** Relative transcript levels of selected genes of *CA_P0037::int* mutant in the three metabolic states (acidogenesis (AC), alcohologenesis (AL) and solventogenesis (SO)) and of oxygen exposed WT (+O_2_ WT (5)), ΔPerR mutant (5), iron limited WT (-Fe WT (6)) and Fur::int mutant (6). n.a.: not available.

|  |  |  | **Fold regulation** | | | | | | | **Putative binding boxes** | | |
| --- | --- | --- | --- | --- | --- | --- | --- | --- | --- | --- | --- | --- |
| **ORF** | **Gene** | **Protein function** | **Cap0037 ::int MT** | | | **+O_2_ WT** | **ΔPer MT** | **−Fe WT** | **Fur : : int MT** | **Cap37** | **Per** | **Fur** |
|  |  |  | **AC** | **AL** | **SO** |  |  |  |  |  |  |  |
| CAC1027 | *fprA1* | Flavoprotein | 0.97 | 1.06 | 1.01 | 4.4 | 16.1 | n.a. | n.a. | − | + | − |
| CAC2449 | *fprA2* | Flavoprotein | 7.01 | 3.28 | 1.21 | 7.3 | 22.5 | n.a. | n.a. | − | + | − |
| CAC2448 | *nror* | NROR | 6.30 | 2.54 | 1.12 | 7.8 | 16.8 | n.a. | n.a. | − | + | − |
| CAC2450 | *dfx* | desulfoferrodoxin, superoxide reductase | 2.39 | 1.11 | 0.39 | 18.4 | 29.6 | n.a. | n.a. | − | + | − |
| CAC2777 |  | Glutaredoxin | 7.20 | 3.45 | 1.62 | 3.5 | 6.0 | n.a. | n.a. | + | + | − |
| CAC2778 | *rd* | Rubredoxin | 8.47 | 4.30 | 2.00 | 5.2 | 14.4 | n.a. | n.a. | + | + | − |
| CAC3306 | *tpx* | Thiolperoxidase | 2.16 | 0.77 | 0.63 | 3.0 | 3.4 | n.a. | n.a. | − | + | − |
| CAC3597 | *rbr3B* | Reverse rubrerythrin | 1.69 | 0.78 | 1.45 | 5.6 | 26.9 | n.a. | n.a. | − | + | − |
| CAC0447 | *feoB* | Feo protein, involved in Fe^2+^ transport | 18.67 | 70.71 | 5.65 | 2.3 | 1.22 | n.a. | n.a. | − | − | − |
| CAC0448 | *feoB* | Fe^2+^ transport protein B | 6.58 | 17.92 | 2.71 | 2.8 | 1.34 | 3.76 | n.a. | − | − | − |
| CAC0788 |  | Ferrichrome transport permease | 76.51 | 32.37 | 33.6 | 3.3 | n.a. | 207 | 33.6 | − | − | + |
| CAC0789 | *fhuB* | Permease | 125.0 | 50.73 | 59.5 | 3.4 | n.a. | 199 | 37.4 | − | − | + |
| CAC0790 | *fhuD* | Ferrichrome-binding periplasmic protein | 195.4 | 128.3 | 50.7 | 2.6 | n.a. | 86.7 | 54.4 | − | − | + |
| CAC0791 | *fhuC* | Ferrichrome ABC transporter, ATP-binding protein | ∞ | 90.69 | ∞ | 2.3 | n.a. | 122.2 | 67.0 | − | − | + |
| CAC1029 | *feoA* | FeoA-like protein, involved in iron transport | 195.6 | 243.3 | 76.2 | 5.8 | 0.99 | 100.5 | 140.5 | + | − | + |
| CAC1030 | *feoA* | FeoA-like protein, involved in iron transport | 252.1 | 283.6 | 83.1 | 8.1 | 1.06 | 113.8 | 154.7 | + | − | + |
| CAC1031 |  | FeoB-like GTPase, responsible for iron uptake | 220.9 | 337.1 | 68.6 | 15.9 | 1.13 | 246.7 | 235.9 | + | − | + |
| CAC1032 |  | Predicted transcriptional regulator | 318.1 | 452.7 | 82.4 | 4.3 | 0.94 | 218.1 | 159.9 | + | − | + |
| CAC0587 | *fld1* | Flavodoxin | 461.9 | 1197 | 119 | 11.4 | n.a. | 275.7 | 312.4 | − | − | + |
| CAC2452 | *fld* | Flavodoxin | 6.15 | 3.90 | 1.58 | 3.6 | 11.6 | n.a. | n.a. | − | + | − |
| CAC0590 | *ribD* | Pyrimidine deaminase and pyrimidine reductase | 8.13 | 1.78 | 3.36 | 2.08 | 1.22 | 65.4 | 78.9 | − | − | + |
| CAC0591 | *ribB* | Riboflavin synthase subunit α | 9.07 | 1.31 | 3.68 | 1.72 | 1.1 | 55.3 | 57.2 | − | − | + |
| CAC0592 | *ribA* | GTP cyclohydrolase/3,4-dihydroxy-2-butanone 4-phosphate synthase | 10.83 | 1.30 | 3.56 | 1.5 | 1 | 44.2 | 61.9 | − | − | + |
| CAC0593 | *ribH* | 6,7-dimethyl-8-ribityllumazine synthase | 5.88 | 1.02 | 1.98 | 1.71 | 1.05 | 41.0 | 49.0 | − | − | + |

References

1. **Hillmann F**, **Döring C**, **Riebe O**, **Ehrenreich A**, **Fischer R-J**, **Bahl H**. 2009. The role of PerR in O2-affected gene expression of *Clostridium acetobutylicum*. J Bacteriol **191**:6082–93.

2. **Vasileva D**, **Janssen H**, **Hönicke D**, **Ehrenreich A**, **Bahl H**. 2012. Effect of iron limitation and fur gene inactivation on the transcriptional profile of the strict anaerobe *Clostridium acetobutylicum*. Microbiol (United Kingdom) **158**:1918–1929.
